# Does the source of funding matter?

**DOI:** 10.7196/AJTCCM.2018.v24i1.208

**Published:** 2018-04-03

**Authors:** Richard van Zyl-Smit, Jonathan Grigg

**Affiliations:** 1 University of Cape Town Lung Institute and Division of Pulmonology, Department of Medicine, University of Cape Town and Groote Schuur Hospital, Cape Town, South Africa; 2 Respiratory and Environmental Medicine, Queen Mary’s Hospital London, Centre for Genomics and Child Health, Blizard Institute, Queen Mary University of London, United Kingdom

## Editorial

In the competitive space of research and clinical medicine, funding is vitally important to maintain services and staff. For many years, the medical profession has battled with conflicts of interest – be they real or perceived, perverse incentives and many cases of downright unethical kickbacks. Ben Goldacres’ book *Bad Pharma* is an eye-opening and exhaustive account of how ‘who pays the bills’ matters. We have rightly seen a tightening of regulations regarding industry funding of events, and ‘educational talks’ in far-off luxury resorts are a thing of the past. These industry relationships have clearly established boundaries and, for publications and presentations, obligatory declared conflicts of interest allow the audience to decide on the scientific merits and potential undue influence by the funder on the researcher/clinician.

The tobacco industry, however, provides a far more complex relationship to negotiate. In addition to producing commercial products that potentially kill the user, it is well recognised that the industry has spent vast sums of money on obstructing tobacco control regulations, producing favourable research outputs and actively marketing tobacco to the youth for many years. This approach is veiled as protecting their intellectual property, but effectively protects and increases their revenue. ‘Harm reduction’ is the new buzz phrase in and around tobacco smoking, with ‘heat-don’t-burn’ products being marketed along with electronic cigarettes as a ‘safer alternative to smoking’. Harm reduction is vitally important in all aspects of medical care – from diabetes to HIV – but the cynic will argue that tobacco companies also believe in harm reduction. Based on their website statements, they do, but this is not out of apparent concern for the public, and is rather to keep their clients alive for longer so that they can spend more money buying cigarettes.

So, does the funding source matter? The newly established Foundation for a Smoke-free World, lead by a former World Health Organization (WHO) anti-tobacco champion, Dereck Yach, is offering independent research grants to the medical fraternity. However, the money is primarily provided by Phillip Morris International, which is a leading international tobacco company. Both parties profess to have no influence on the outcomes of the research funding or results. So, does it matter who gave the money?

Both the American Thoracic Society (ATS) and the European Respiratory Society (ERS) have previously instituted a rigid policy that no funding would be accepted from a tobacco company source, and no research funded by such a source would be presented or published in their meetings/journals. The policy also extends to organisations that are primarily funded by tobacco money, such as the Foundation for a Smoke-free World. The WHO opposes this foundation and its funding, as it contravenes the Framework Convention on Tobacco Control (FTCT) through the involvement of the tobacco industry in healthcare matters. The ATS and ERS have taken a further ethical stance – receiving money from a company that is responsible for direct harm to respiratory patients is not acceptable, as the primary mandate of the societies is the promotion of respiratory health. Similarly, the South African Thoracic Society (SATS) does not accept funding from tobacco sources and, although this new funding may be considered independent, its source is the very lives of patients with tobacco-related illness whom we care for. SATS has taken a stance that is aligned with the ERS and ATS and which may be seen by some to be short-sighted and taking a false moral high-ground. As pulmonologists, money that has been generated by causing the very illnesses our patients suffer and die from, is money we should live without.

